# Non-Invasive and Mechanism-Based Molecular Assessment of Endometrial Receptivity During the Window of Implantation: Current Concepts and Future Prospective Testing Directions

**DOI:** 10.3389/frph.2022.863173

**Published:** 2022-05-04

**Authors:** Bei Sun, John Yeh

**Affiliations:** ^1^Sackler Faculty of Medicine, Sackler School of Medicine, New York State/American Program of Tel Aviv University, Tel Aviv University, Tel Aviv, Israel; ^2^Reproductive Endocrinology and Infertility, UMass Memorial Medical Center, University of Massachusetts Medical School, Worcester, MA, United States

**Keywords:** endometrial receptivity, window of implantation (WOI), mechanism, molecular markers, point-of-care testing, non-invasive testing

## Abstract

Suboptimal endometrial receptivity and altered embryo-endometrial crosstalk account for approximately two-thirds of human implantation failures. Current tests of the window of implantation, such as endometrial thickness measurements and the endometrial receptivity assay, do not consistently improve clinical outcomes as measured by live birth rates. Understanding the mechanisms regulating the endometrial receptivity during the window of implantation is a critical step toward developing clinically meaningful tests. In this narrative review, the available literature is evaluated regarding mechanisms that regulate the endometrial receptivity during the window of implantation and the current tests developed. Overall, both animal and human studies point to five possible and interrelated mechanisms regulating the endometrial window of implantation: suitable synchrony between endometrial cells, adequate synchrony between the endometrium and the embryo, standard progesterone signaling and endometrial responses to progesterone, silent genetic variations, and typical morphological characteristics of the endometrial glands. The biological basis of current clinical markers or tests of window of implantation is poor. Future studies to elucidate the mechanisms shaping the window of implantation and to investigate the potential markers based on these mechanisms are required. In addition, molecular testing of the endometrium at single-cell resolution should be an initial step toward developing clinically meaningful tests for the optimal window of implantation. As understanding of the optimal window of implantation continues to evolve, one can envision the future development of non-invasive, mechanism-based testing of the window of implantation.

## Introduction

Early pregnancy loss is common in women, with only 30% of conceptions reaching live birth. Establishing a successful pregnancy depends upon the implantation. Suboptimal endometrial receptivity and altered embryo-endometrial crosstalk account for approximately two-thirds of implantation failures. Understanding the causes of implantation failures has been identified as one of the top 10 priorities of future infertility research (1). Implantation is a highly organized process during which the embryo attaches to the surface of the endometrium, invades the epithelium, and then the maternal circulation to form the placenta. Synchronous structural and functional remodeling of the uterine endometrium and the blastocyst is essential for successful implantation (2, 3). The window of implantation (WOI) is a limited time span during which crosstalk between a receptive uterine endometrium and a competent blastocyst occurs effectively (4, 5). For a normal 28-day menstrual cycle, WOI usually occurs between days 19 and 21 of the cycle (6). During the WOI, structural and molecular conditions of the endometrium and the surrounding environment allow euploid embryos to implant (7). Despite past and current efforts at characterizing the WOI, the mechanisms regulating the WOI are not clearly understood. This article reviews current literature on the mechanisms shaping the receptivity of endometrium during the WOI as well as markers used to assess WOI. Possible future directions for developing non-invasive and mechanism-based testing of the WOI are outlined.

## Search Method

A total of 465 articles were reviewed. For the section on mechanisms regulating the WOI, the authors performed a PubMed search using the following keywords: window of implantation, endometrium, and mechanism. No time limits were placed at the time of publication and 134 results were obtained. Animal and human studies as well as references from review articles that discuss pathways influencing the WOI were selected. For the section on current tests of the WOI, the authors performed another PubMed search using the following keywords: window of implantation and clinical test. No time limits were placed at the time of publication and 331 results were obtained. Clinical studies that report non-molecular and molecular markers of the WOI were selected.

## Potential Mechanisms Regulating the Optimal WOI

**Figure 1:**
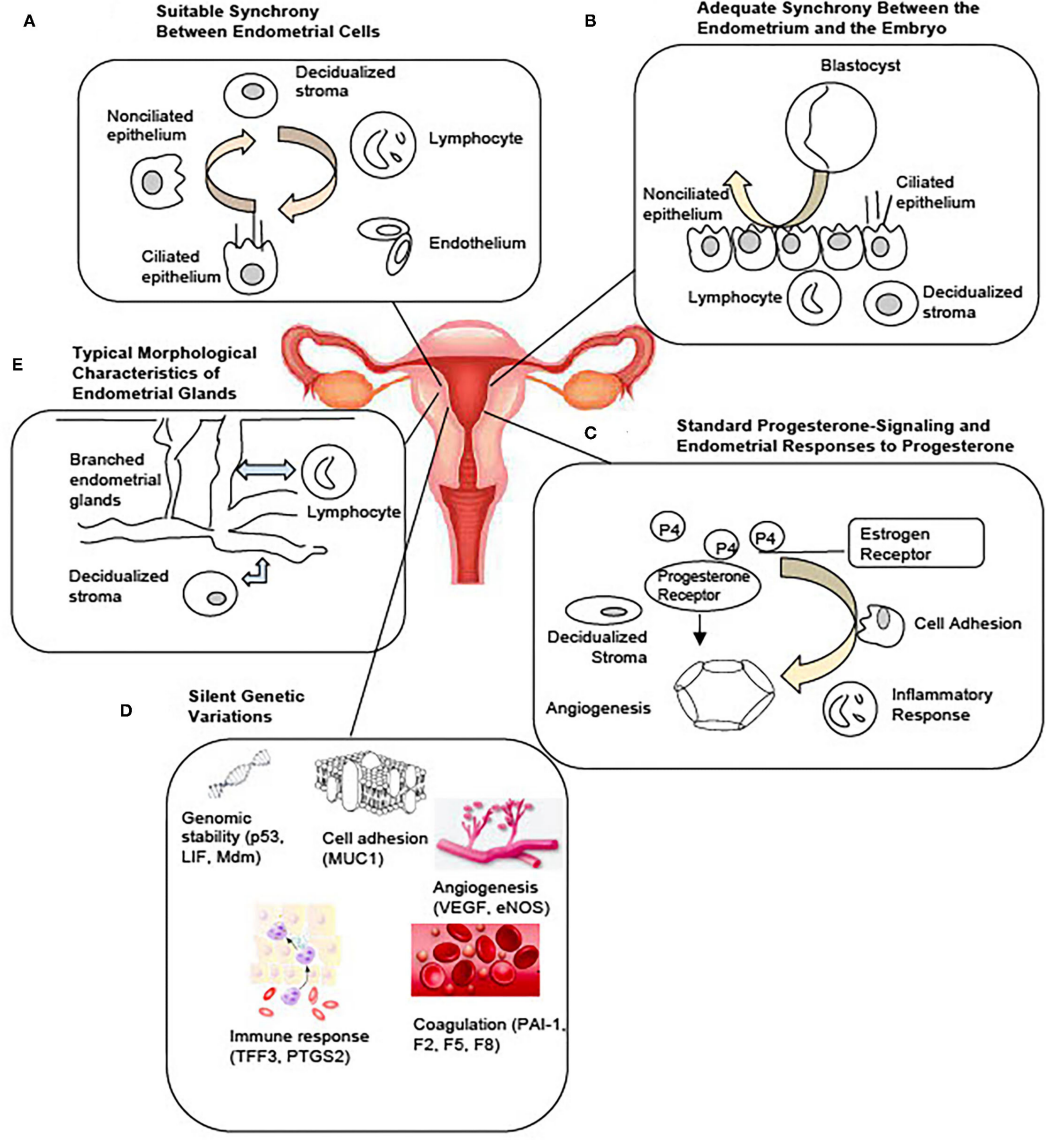
Potential Mechanisms Regulating the Optimal Window of Implantation. Based on current literature, five possible and interrelated mechanisms include: **(A)** Suitable synchrony between the endometrial cells. **(B)** Adequate synchrony between the endometrium and the embryo. **(C)** Standard progesterone-signaling and the endometrial responses to progesterone. **(D)** Silent genetic variations. **(E)** Typical morphological characteristics of the endometrial glands may together constitute the molecular basis of a receptive endometrium during the window of implantation.

Factors influencing endometrial development and embryo implantation have long been investigated and the concept of endometrial receptivity during the WOI is not new. Recent molecular studies have enhanced our understanding of the complex process of implantation and led to the development of commercial tests for the evaluation of the endometrium (8). Yet, the molecular mechanisms regulating the WOI are not completely understood. The diagnostic value of the existing clinical tests is not established. Based on the current understanding of the endometrium and the implantation process, the following are the probable and interrelated five mechanisms regulating a receptive endometrium during the WOI (Figure 1): (1) Suitable synchrony between endometrial cells; (2) Adequate synchrony between the endometrium and the embryo; (3) Standard progesterone-signaling and endometrial responses to progesterone; (4) Silent genetic variations; and (5) Typical morphological characteristics of the endometrial glands. Disturbances in a single mechanism or a combination of the mechanisms would be predicted to result in suboptimal endometrial receptivity during the WOI, which then results in suboptimal clinical outcomes.

### Suitable Synchrony Between Endometrial Cells

Entry into WOI requires a synchronous molecular programming process between the endometrial cells across the entire endometrium (Figure 1A). Asynchronous molecular activities between the endometrial cells can lead to a suboptimal environment for the embryo to implant. A recent single-cell RNA profiling of the human endometrium throughout the menstrual cycle identified six distinct cell types, such as stromal fibroblasts, macrophages, lymphocytes, and ciliated epithelial, non-ciliated epithelial, and endothelial cells (9). A unique transcriptomic signature during the WOI for each cell type was reported. Molecular synchrony between endometrial cells of the same lineage as well as between endometrial cells of different lineages may be a determining factor that shapes the WOI. Synchronous epigenetic and transcriptomic reprogramming in endometrial cells facilitates local and global morphological as well as biochemical changes essential to entering and maintaining the WOI. For example, the process of decidualization was characterized by the interplay of stromal fibroblasts, lymphocytes, epithelial, and endothelial cells. Modular upregulation of decidualization-initiating factor FOXO1 and other genes, such as DKK1 and CRYAB, in stromal fibroblasts occurs in sync with upregulation of CD69, ITGA1, and CD56 expression in surrounding lymphocytes and with downregulation of CXCL14, MAOA, DPP4, and metallothionein as well as upregulation of MMP7 and THBS1 in unciliated epithelial cells. Other synchronous molecular changes between different endometrial cell lineages essential to the WOI can be further characterized. Synchronous molecular activities between endometrial cells of the same lineage may be equally important to the WOI. Future studies are necessary to identify these essential synchronous molecular events and to characterize the extent to which each event contributes to the entry, maintenance, and exit of the WOI.

Coordinated molecular and structural changes of endometrial cells sustain the morphology, plasticity to respond to environmental cues, and biosynthesis needs underlying a receptive endometrium. Such molecular synchrony needs to be maintained at potential implantation sites across the entire endometrium to optimize the window of implantation. In mice, implantation crypts arise from the epithelial evaginations with pre-existing glands, which allow direct communication between the glands and the implanted embryo (10). Inter-implantation sites consist of a high density of glands. This topography suggests that potential implantation sites interspersed by dense glandular tissues populate the endometrium. Different synchronous activities between a distinct set of endometrial cell lineages may be maintaining the implantation and inter-implantation sites. Whether a similar topography for implantation applies to the human endometrium remains to be investigated. Current tests of WOI, such as the endometrial receptivity assay (ERA), that obtain samples from one area of the endometrium do not necessarily capture the molecular activities of the potential implantation sites. In addition, such tests examine the static molecular states and also do not report gene expression at the cellular level. As a result, they do not assess for molecular synchrony among the endometrial cells. Future studies should investigate the mechanisms through which synchronous molecular programming in endometrial cells is disrupted to establish a threshold of asynchrony above which implantation cannot occur.

### Adequate Synchrony Between the Endometrium and the Embryo

Synchronous molecular activities for appropriate communication between the endometrium and the embryo are essential components of endometrial receptivity during WOI (Figure 1B). Asynchronous molecular activities between the endometrium and the embryo disrupt signaling cascades necessary for a successful implantation event. With the development of egg freezing techniques and egg donation programs, oocyte maturation and induction of endometrial receptivity are often dissociated and assessed separately. Studies in the 1990's suggested that a critical window in the menstrual cycle determines whether implantation will or will not occur (6, 11, 12). One study showed that detection of urinary human chorionic gonadotropin (hCG) occurred from day 8–10 post-ovulation in 84% of the successful pregnancies (11). The result suggests that the optimal implantation rates occur with embryo-endometrial asynchrony of ± 1.5 days or less. Embryos transferred between days 17 and 19, with embryo-endometrial asynchrony of < ±1.5 days led to a pregnancy rate of 32.4% while embryos transferred before or after this time frame did not lead to implantation in a study of oocyte donation cycles in women with ovarian failure (6). A more recent study investigated the effect of day 5 and day 6 blastocyst transfers on implantation and pregnancy rates between fresh autologous cycles and frozen embryo transfer cycles (13). In fresh autologous cycles, the clinical pregnancy rate was higher for day 5 blastocyst transfers than for day 6 blastocyst transfers while no significant difference was found between transfers of blastocysts cryopreserved on day 5 and day 6 in frozen embryo transfer cycles. Similar embryo-endometrial synchrony between day 5 and day 6 blastocyst in frozen embryo transfer cycles leads to similar clinical outcomes as ovarian stimulation with pituitary suppression is known to induce a more histologically advanced endometrium than in natural cycles (14, 15).

The communication between the embryo and the endometrium during the WOI is essential to the success of the implantation (Figure 1B). Shahbazi et al. reported that the critical remodeling events, such as generation of a bi-laminar disc, formation of a pro-amniotic cavity, appearance of a prospective yolk sac, and trophoblast differentiation at implantation, are embryo-autonomous (16). This highly self-contained molecular programming of early implantation embryo needs to occur in synchrony with the molecular programming of the endometrial cells. Studies have reported a bi-directional exchange of extracellular vesicles between the endometrial cells and the embryo throughout the process of implantation (17–19). The extracellular vesicles may be one of the important channels that facilitate synchronous molecular programming between the endometrium and the embryo. The molecular content within the extracellular vesicles and its role in maintaining the molecular synchrony requires future investigations. As synchronous molecular changes between the endometrial cells occur, leading up to a receptive state, continued molecular synchrony between the endometrial cells as well as the embryo may be essential to successful sustained implantation.

### Standard Progesterone-Signaling and Endometrial Responses to Progesterone

Appropriate progesterone signaling and endometrial responses are critical to endometrial receptivity (Figure 1C). Dysfunctional progesterone signaling leading to abnormal endometrial changes significantly disrupts the WOI. Progesterone was first discovered for its effects on the endometrium and early pregnancy survival (20, 21). It is essential in the successful embryo implantation and pregnancy. Progesterone production of insufficient quantity (<5 ng/ml during the luteal phase) or temporal duration (luteal phase duration <10 days) and inadequate endometrial response to adequate progesterone levels have been described as components of the pathophysiology of the luteal phase deficiency associated with pregnancy-related disorders, such as infertility and early pregnancy loss (22). A study of controlled cycles with a protocol that started with gonadotropin hormone-releasing hormone (GnRH) agonist downregulation, followed by transdermal estrogen replacement at physiological concentrations and daily intramuscular progesterone at physiological and sub-physiological concentrations investigated the effect of exposure to different progesterone concentrations on endometrial function (23). The study found no difference in histological dating of endometrium, immunohistochemistry for endometrial integrins, and expression of nine putative functional products, despite a 4-fold difference in progesterone exposure between the groups. The result suggests that normal secretory-phase endometrial structure and function can be achieved through a wide range of progesterone concentrations and that no minimum serum progesterone concentration defines normal or fertile luteal function. Histologic dating of the endometrium with endometrial biopsy, however, is no longer recommended as a number of factors beyond the histology, such as steroid receptors, structural proteins, pinopodes, growth factors, and cytokines, is associated with implantation (24–28).

Another proposed pathophysiology underlying luteal phase deficiency implicated in suboptimal WOI is an inadequate endometrial response to adequate progesterone levels or progesterone resistance. Endometriosis is an inflammatory condition that has been associated with progesterone resistance. The inflammatory signals, such as IL-6 and IL-17, contribute to the elevation of kristen rat sarcoma viral oncogene (KRAS) and prolonged phosphorylation of signal transducer and activator of transcription 3 (STAT3) which stabilizes the hypoxia-induced factor and vascular endothelial growth factor (VEGF) pathways normally expressed only at menstruation (29, 30). KRAS and phosphorylated STAT3 also activate Sirtuin-1 (SIRT1) and BCL6 which are key mediators implicated in the progesterone resistance (31). SIRT1 is a histone deacetylase that inactivates signaling pathways downstream of the progesterone receptor (29). SIRT1 and BCL6 target the COUP-TFII/Indian Hedgehog pathway implicated in the progesterone signaling (29, 32). Progesterone receptor dysfunction also contributes to progesterone resistance. PR-A and PR-B are two isoforms of progesterone receptors that mediate the downstream signaling. Altered ratios of PR-A and PR-B expressions have been associated with the development of progesterone resistance (33). In addition, the upregulation of aromatase and increased level of estrogen contribute to the dysregulation of progesterone signaling (34). The development of progesterone resistance and its effects on the WOI are not clearly understood. Dysfunctional progesterone signaling may disrupt the molecular synchrony between the endometrial cells necessary for effective communication with competent embryos. Future studies should investigate the effects of progesterone on the dynamics of transcriptomic changes in each endometrial cell lineage.

### Silent Genetic Variations

Non-pathological genetic variants implicated in processes, such as angiogenesis, immune response, coagulation, cell adhesion, and genome stability, are important in establishing the WOI (Figure 1D). Pathological genetic variants can lead to dysregulation of critical processes during the implantation of embryos. A few variants have been reported so far. Genetic variants regulate the process of angiogenesis that influence the development of a vascular network during implantation, embryo development, and placentation. VEGF is a potent angiogenic factor. Four VEGF polymorphisms that affect VEGF activity and expression have been associated with recurrent pregnancy loss (RPL), spontaneous abortion, and implantation failure (35–38). Endothelial nitric oxide synthase (eNOS), expressed in terminal chorionic villous vessels, produces vascular nitric oxide to supply nutrients to the fetus. One eNOS polymorphism has been associated with RPL (39). Genetic variants of thrombolytic factors, such as plasminogen activator inhibitor-1 (PAI-1) and coagulation factors II, V, and VIII (F2, F5, and F8), have also been associated with early pregnancy loss (40, 41). Processes involved in the immune and inflammatory response play an important role in successful implantation. Genetic variants of prostaglandin-endoperoxide synthase 2 (PTGS2) which converts arachidonic acid to prostaglandins and trefoil factor 3 (TFF3), which promotes epithelial cell migration and mediates endothelial repair, have been associated with an increased risk of implantation failure (42, 43). In addition, single nucleotide polymorphisms in the p53 pathway have been associated with implantation failure (44). Cell adhesion molecules are essential to the implantation process. Low levels of mucin 1 (MUC1), an anti-adhesion molecule secreted by the human, have been associated with recurrent implantation failure and a small allele size of MUC1 has been found in women with unexplained infertility (45, 46). Currently, most studies of genetic variants implicated in implantation failure are small association studies. The genetic basis of implantation failure remains an important area that needs to be explored in-depth by future large-scale clinical studies. These small association studies revealed the possibility of identifying additional functional pathologic variants implicated in the processes known to be important in implantation. In addition, novel pathways or processes may be uncovered through genetic investigations of implantation failure. With future studies to advance our understanding of the genetic basis of implantation, genetic variants may be identified through prenatal blood tests to recognize patients at high risk of infertility or pregnancy loss that require early interventions. Targeted therapeutics can be developed based on the understanding of the pathways involved in the variant genes associated with implantation failure.

### Typical Morphological Characteristics of the Endometrial Glands

Characteristics of the morphology of endometrial glands may play an essential role in regulating the receptivity of the endometrium. Deviations from standard characteristics of the endometrial glands can disrupt the WOI (Figure 1E). The importance of endometrial glands in successful implantation has been established for decades (47–49). It has been hypothesized that the glands transport and secrete factors into the endometrial lumen that regulate the growth and survival of the pre-implantation embryo (50, 51). Recent studies suggest that the endometrial glands communicate with many different cell types in the endometrium and may play a significant role in shaping the endometrial receptivity through paracrine interactions (52–54). Morphological features of endometrial glands influence the accessibility and the traffic of the paracrine interactions with the endometrial cells. Three-dimensional characterization of the endometrium revealed a horizontally expanding plexus morphology of the basal glands, an abundance of occluded glands, a higher proportion of branched glands in the secretory phase than in the proliferative phase with a majority of branched glands at the bottom of the endometrium, and a third of the glands sharing branches with other glands (55). Alterations in these morphological features may disturb the reliable, rapid regeneration of the functional layer during each menstrual cycle as well as the synchronous communication between the endometrial cells.

Chronological female age is an important variable in the status of the endometrium. The morphology of the endometrial glands alters with age. For example, the proportions of occluded glands and branched glands correlate with age (55). These changes in the morphology of the endometrial glands with age may have both molecular and mechanical implications on the receptivity of the endometrium. For instance, the increase in occluded glands and branched glands may interfere with the paracrine interactions among the endometrial cells. This interference may lead to increased molecular asynchrony in the endometrium. In addition, the increased number of branched glands may interfere mechanically with the formation of implantation crypts as glands are pushed to the periphery and populate the inter-implantation sites observed in the animal study (10). Accompanying the morphological changes, molecular alterations also occur in the endometrium with age. A recent transcriptomic study of the endometrium of women over 35 and women under 35 years of age identified thousands of differentially expressed genes and noted a down-regulation of epithelial cell proliferation as well as an up-regulation of cilia-related processes (56). The mechanisms through which age-related morphological and molecular changes in the endometrium influence the endometrial receptivity in the WOI should be investigated further as it may contribute to the identification and optimization of implantation. Recent studies have identified distinct features of the endometrial glands during optimal WOI and the potential functional significance of these features. The age-related changes of these features provided additional support for its significance in implantation. A better understanding of the molecular mechanisms regulating the morphological characteristics of the endometrial glands can help us to identify biomarkers indicative of suboptimal WOI for diagnostics.

## Current Clinical Tests for Endometrial WOI

Accurate assessment of the WOI for each woman in the cycle in which they are attempting to implant an embryo spontaneously, by intrauterine insemination (IUI) or by assisted reproductive technology (ART) may significantly improve the pregnancy rates and clinical outcomes. Non-molecular and molecular markers for the WOI have long been evaluated. Yet so far, the clinical value of these currently used markers is still being debated and none of them has been proven to be fully predictive of live birth as an outcome in routine clinical use.

### Non-molecular Markers for the WOI

Historically, histological changes of the endometrium during the secretory phase have been quantified using morphometric methods and the Noyes criteria used as standards to date the endometrium (57). Non-molecular assessment of the WOI nowadays relies heavily on ultrasound imaging. Ultrasound measurements, such as endometrial thickness, volume, pattern, contraction, and blood flow, have been evaluated for their predictive value for the receptivity of the endometrium during the WOI. Among these, endometrial thickness was the most commonly investigated marker and reported at various time points during IUI and *in-vitro* fertilization (IVF) cycles with fresh or non-fresh embryo transfer (58). For IUI cycles, it has been reported during the ovarian stimulation, on the day of hCG injection, and on the day of IUI (59–62); for IVF cycles with fresh embryo transfer, it has been reported during the mid-luteal phase in the cycle preceding the IVF cycle, the day of hCG injection, the day after hCG injection, the day of oocyte retrieval, and the day of embryo transfer (63–70); for IVF cycles with non-fresh embryo transfer, it has been reported on the day of LH surge in the natural cycle, the day of initiating progesterone, and the day of embryo transfer (71–74). While endometrial thickness was reported to be higher on the day of hCG injection in groups that achieved clinical pregnancy than in groups that did not achieve clinical pregnancy in some studies, the result did not correlate with the live birth rates (62, 63, 67, 70, 75). A recent randomized controlled study of 868 couples with unexplained infertility concluded that the endometrial thickness was not independently a reliable determinant of live birth (76). In addition, when cut-offs for the endometrial thickness ranging between 6 and 17 mm measured on the day of hCG injection were employed for women undergoing IVF cycle with fresh embryo transfer, the predictive accuracy of endometrial thickness for the clinical pregnancy was low (58). In the past, clinicians have relied on ultrasound measurements, most commonly the endometrial thickness and the presence of a triple-line pattern to screen for the status of the endometrium. Yet, the connection between these ultrasound measurements and the mechanisms regulating the endometrial receptivity remains to be established. Furthermore, future studies are necessary to elucidate the impact of endometrial thickness on the live birth outcome (77). Studies investigating other non-molecular markers obtained from endometrial fluid aspirate and hysteroscopy currently have not provided sufficient data for the development of markers predictive of clinical pregnancy or live birth (78–82).

### Currently Available Molecular Markers of the WOI

**Figure 2:**
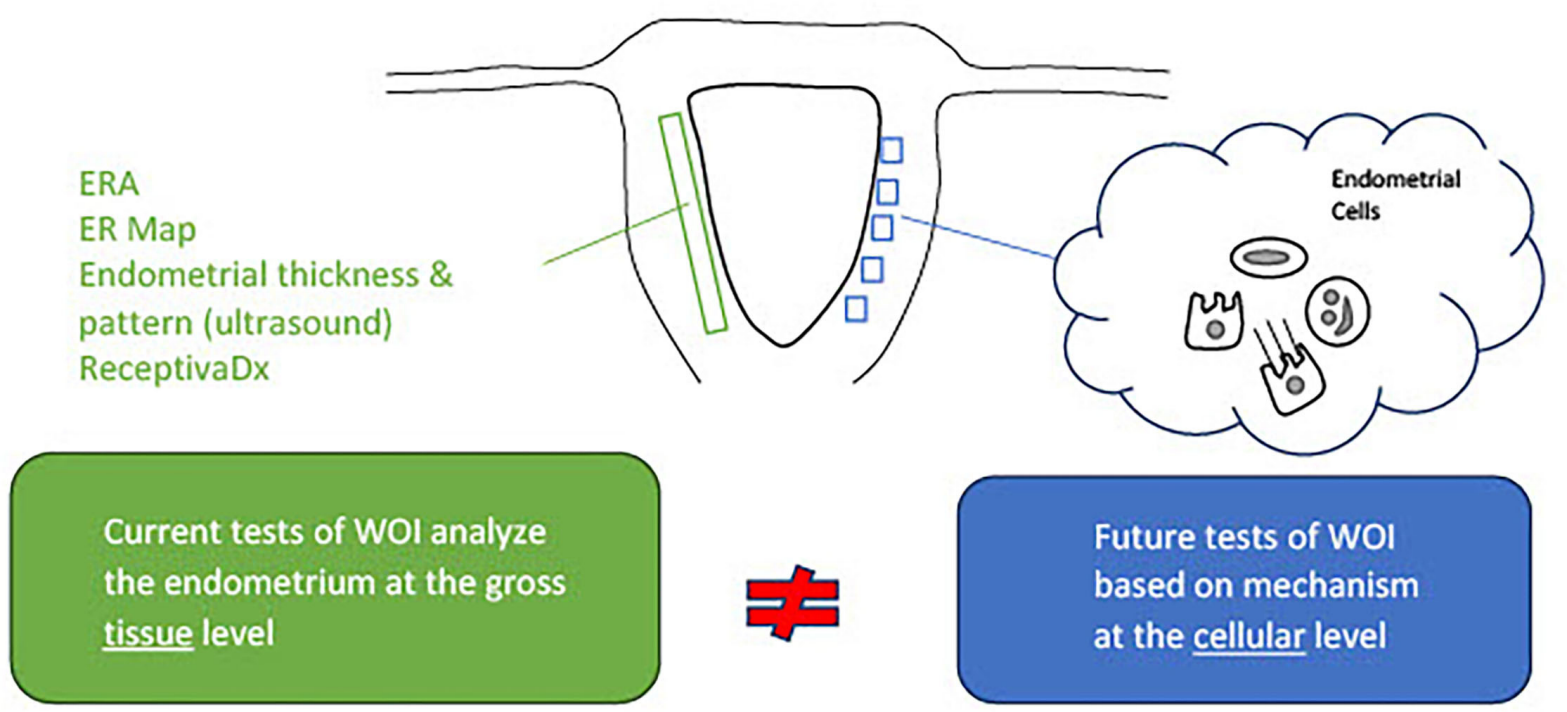
Current tests of the window of implantation are not based on full knowledge of the mechanisms regulating the endometrial receptivity. Activities of the endometrial cells underly the processes essential to the entry and maintenance of the window of implantation. Commercial tests of window of implantation, however, do not analyze the endometrium at the cellular level necessary to investigate the status of receptivity.

Molecular markers of the WOI have long been investigated. They provide insights into the biological processes crucial to the successful implantation and hold the potential for functional assessment of the endometrium. Commercial molecular tests, such as the endometrial receptivity assay (ERA) and the endometrial receptivity (ER) map, utilize genes reported to be implicated in the window of implantation. The ERA is a microarray-based technology that examines the endometrial expression of 238 genes and classifies the endometrium accordingly into receptive, pre-receptive, or post-receptive categories (83). Its value in improving implantation rates, however, has not been proven (84–86). The ER Map quantitatively assesses the expression of 40 genes implicated in pathways, such as cell proliferation and division and immunological activity, that were found to be differentially expressed in the receptive and non-receptive endometrial samples (87). Another commercial test, ReceptivaDx, measures the expression of BCL6 which is implicated in progesterone resistance. A clinical study has linked a high level of BCL6 to a poor IVF outcome (88). Yet, similar to ERA, its clinical value has not been proven. Various individual markers have also been reported with insufficient data to support their clinical value (45, 88–90). With the development of omics technology, transcriptomic, proteomic, and lipidomic profiling of the endometrium could be performed. Differential expressions of nucleic acid, protein, and lipid markers during the mid-secretory phase and the proliferative phase have revealed potential pathways implicated in the regulation of endometrial receptivity and have been evaluated as potential markers of WOI. Potential non-invasive molecular markers have also been reported. Recent studies have identified microRNA markers associated with the recurrent implantation failure in blood and uterine fluid (91, 92). Additional studies may reveal whether they could serve as possible non-invasive receptivity markers. A comprehensive review of the markers of endometrial receptivity has previously been performed. Selected studies illustrate the variety of molecule types and sources of sample collection, reported in Table 1. While studying a variety of molecule types from different sources may reveal the novel pathways involved in endometrial receptivity, this approach is problematic in advancing the development of clinically meaningful markers. The limitations of these current studies are highlighted and need to be addressed. First of all, the markers have been reported from different sources, such as endometrial tissue from biopsies or endometrial fluid. Source-specific markers need to be identified. Another limitation is that these molecular markers do not detect asynchrony between the endometrial cells. While these markers may reflect global changes in the molecular activities of the endometrium; markers from single-cell analysis would capture the disturbances in the communication between endometrial cells and more accurately reflect the receptivity of the endometrium (Figure 2).

**Table 1:** Currently described molecular markers of human endometrial window of implantation.

**Molecule**	**Source**	**Examples of Biomarkers**	**Associated Pathway**	**Results**	**Reference**
mRNA	Endometrial tissue biopsy	PAEP, SPP1, GPX3, MAOA, GADD45A, SFRP4, EDN3, OLFM1, CRABP2	Responses to external stimuli and wounding; inflammatory responses; coagulation; immune responses	52 genes were upregulated and 5 genes were downregulated at mid-secretory endometrium compared to pre-receptive endometrium	(93)
microRNA	Serum	miR-200c	miR-200c inhibits FUT4 and α-1,3-fucosylation biosynthesis, particularly on CD44. Fucosylation of uterine endometrium mediates recognition and adhesion at the fetal-maternal surface	miR-200c was decreased in serum of pregnant women compared with that in healthy non-pregnant women, and increased in infertility and abortion patients	(94)
	Blastocyst culture media	miR-661	miR-661 downregulates PVRL1 mRNA and protein in primary endometrium epithelial cells, which impairs epithelial cell polarity in endometrial luminal epithelia and adhesion	miR-661 was secreted by blastocysts that failed to implant	(95)
	Endometrial biopsy	miR-181a	miR-181a downregulates KLF12 mRNA and protein, a transcription factor critical to the differentiation of stromal cells and decidualization	miR-181a increased significantly during induced decidualization. KLF12 overexpression abolished miR-181a induced decidualization.	(96)
EV-sncRNA^*^	Endometrial fluid (endometrial exosome vesicles)	miR-449c-5p, miR-497-5p, miR-767-5p, miR-501-5p, miR-411-3p, miR-196a-5p, miR-4636, miR-18a-5p, miR-493-5p, miR-503-5p, miR-671-5p piR-456	Immune response, extracellular matrix, cell junction	11 microRNA and 1 piwi-interacting RNA were differentially expressed between LH+2 and LH+7, and between hCG+2 and hCG+7 groups.	(97)
Protein	Endometrial fluid	PGR, NNMT, SLC26A2, LCN2	PGR: progesterone resistance NNMT: cellular migration/invasiveness, tumor development SLC26A2: sulfate ion transporter induced by progesterone LCN2: neutrophil gelatinase-associated lipocalin	367 proteins underwent significant proteomic changes while transitioning from early-secretory (ESE) to mid-secretory endometrium (MSE). A panel of PGR, NNMT, SLC26A2 and LCN2 achieved a sensitivity and specificity of 91.7% for distinguishing MSE from ESE.	(98)
Lipid	Endometrial fluid	ratio of triacylglycerol and phospholipids	Progesterone signaling, steroid metabolism	Fifteen ion ratios, 13 hyper-represented in the negative implantation group and 2 hyper-represented in the positive implantation group were used to build an ROC curve with AUC of 84% for prediction of endometrial receptivity.	(99)

* *extracellular vesicle derived small non-coding RNA*.

## Proposed Directions to Develop Tests for Optimal Endometrial WOI

### Approaches to Developing Tests for the WOI

Current non-molecular and molecular markers used to assess the endometrial receptivity during the optimal WOI are not developed based on an understanding of the mechanism of the implantation process (Figure 2). Rather, they are derived from the relatively limited studies that demonstrated differential clinical outcomes using these markers. A rigorous approach of developing and validating tests for optimal WOI requires the discovery of markers, correlation of markers with the status of the endometrium, and clinical outcomes. The discovery of predictive markers of optimal WOI depends on an adequate understanding of the underlying mechanisms for implantation and their relationship to live birth rates.

#### Discovery of New Markers of the WOI

Recent studies have pointed to a few mechanisms discussed in the earlier sections in regulating the WOI. These mechanisms should be considered in the investigation of potential markers of the optimal WOI (Table 2). Specifically, an understanding of normal uterine gland biology and cell–cell communication during the receptive phase of the endometrium can guide the development of predictive markers of the WOI. Future studies are necessary to further this understanding. First of all, studies of transcriptomic profiling at the single-cell level for each type of endometrial cell are essential. Signature transcriptomic profiles of single endometrial cells during the WOI could be established which would advance our understanding of the molecular pathways specific to each cell type implicated in sustaining the WOI. It may be that individual endometrial cells not only have to meet threshold levels of certain gene expressions but also threshold relative expression levels of genes in synchrony with other endometrial cells. The evaluation of the molecular synchrony among endometrial cells involves analyzing the relevant gene expression levels of each endometrial cell type relative to the expression levels of the other endometrial cells. The discovery of these molecular pathways can serve as a guide to further study the differential expression of ligand–receptor pairs in endometrial cells, such as stromal fibroblasts and lymphocytes, during the WOI and other phases of the menstrual cycle. It would reveal critical pathways of communication between the endometrial cells. In addition, metabolomic profiles of endometrial cells during WOI can be established. The goal of these studies would be to identify differential transcriptomic profiles, expression of ligand–receptor pairs, or metabolomic profiles as potential markers of the WOI. Performing endometrial biopsy during this first stage of marker discovery may be necessary as it provides a direct route to collect the endometrial cells. Non-invasive routes of obtaining the markers, such as through blood tests, urine tests, cervical swabs, or saliva tests, could be explored as the biological basis of the markers becomes established. Different sets of markers predictive of endometrial receptivity may exist in blood, urine, saliva, or cervical swab tests. For example, nucleic acid and protein markers reflective of endometrial cell activities may be found in blood tests and cervical swabs while metabolomic markers may be found in urine and saliva tests.

**Table 2:** Potential mechanism-based markers for the development of non-invasive tests for the window of implantation (WOI).

**Marker type**	**Non-invasive future tests to detect in blood, cervical swab, urine and/or saliva**	**Expected findings in optimal WOI**	**Challenges**	**Reference**
**A Possible non-invasive mechanism-based markers for the synchrony between endometrial cells and for between endometrial cells and embryo**.				
mRNA levels	Quantitative measurements: transcription factors (TFs) IRX3, PAX8, MITF, ZBTB20 (early developmental regulators), DDIT3 (endoplasmic reticulum stress), FOS, FOSB, JUN, PAEP, GPX3, CXCL14, MAOA, NUPR1, DPP4 in unciliated epithelial cells TFs BHLHE40, ATF3 (cAMP pathway mediated chondrocyte differentiation, probable drivers for decidualization), YBX3, ZBTB16 (endoplasmic reticulum stress), CEBPD (inflammation), STAT3 (apoptosis), DKK1, CRYAB, FOXO1, IL15 in stromal fibroblasts CD69, ITGA1, CD56 (NK cell features) in lymphocytes IL15, IL2RB IL2RG, MHCI and NKR in stromal fibroblasts and lymphocytes	High expressions of IRX3, PAX8, MITF, ZBTB20, DDIT3, FOS, FOSB, JUN, PAEP, GPX3, CXCL14, MAOA, NUPR1, DPP4 in unciliated epithelial cells High expressions of BHLHE40, ATF3, YBX3, ZBTB16, CEBPD, STAT3, DKK1, CRYAB, FOXO1, IL15 in stromal fibroblasts High expressions of CD69, ITGA1, CD56 in lymphocytes High expressions of ligand-receptor pairs IL15, IL2RB,IL2RG,MHC1 and NKR in stromal fibroblasts and lymphocytes	-Capture of intact endometrial cells from different sources -Technical noise from single-cell RNA sequencing data and batch effect may lead to misinterpretation of data and mask the underlying biology -Integrating single-cell analysis into routine clinical practice	(9)
Protein levels	Quantitative measurements: TNC, DNB1, PAEP, CPM, PALLD, MCM6, ENPP3, PPL, HGD, PIGR in epithelial cells APOC3, TNC, DNB1, MME, COL4A2 in stromal fibroblasts	High expressions of PAEP, CPM, PALLD, MCM6, ENPP3, PPL, HGD, PIGR and low expressions of TNC, DNB1 in epithelial cells High expressions of MME, COL4A2 and low expressions of APOC3, TNC, DNB1 in stromal fibroblasts		(100)
**B Possible non-invasive mechanism-based markers for the progesterone signaling and endometrial responses**.				
mRNA levels	Quantitative measurement of PGR, NR2F2, IHH, HAND2, BMP2, HOXA11, HOXA10, WNT4, GLI1, KRAS, SIRT1, BCL6	High expressions of PGR, NR2F2, IHH, HAND2, BMP2, HOXA11, HOXA10, WNT4, GLI1 Low expressions of KRAS, SIRT1, BCL6	-Markers derived mainly from animal studies	(29)
microRNA levels	Quantitative measurement of miRNA21, miRNA21-5p, miRNA21-3p, miRNA29b, miRNA145, miRNA34 (mouse), miRNA29c (baboon), miRNA199a, miRNA122, miRNA-17-5p, miRNA20a, miRNA22	High expressions of miRNA21, miRNA21-5p, miRNA21-3p, miRNA29b, miRNA34 (mouse),miRNA-17-5p, miRNA20a, miRNA22 Low expressions of miRNA29c (baboon), miRNA199a, miRNA122		(101–103)
Metabolite levels	Quantitative measurements of glycolysis metabolites (pyruvate, fructose-6-phosphate, 1,3-diphosphateglycerate, phosphoenolpyruvate, glyceradehyde-3-phosphate) and pentose-phosphate pathway intermediates (sedoheptulose-1,7-phosphate, erythrose-4-phosphate, ribose-phosphate)	Low levels of glycolysis metabolites (pyruvate, fructose-6-phosphate, 1,3-diphosphateglycerate, phosphoenolpyruvate, glyceradehyde-3-phosphate) and pentose-phosphate pathway intermediates (sedoheptulose-1,7-phosphate, erythrose-4-phosphate, ribose-phosphate)		(104)
**C Possible non-invasive mechanism-based markers for genetic variant expression**.				
DNA polymorphism	Sequence analysis of the following DNA polymorphisms: TP53: rs1042522 LIF: T1414G, rs929271 MDM4: rs1563828 MDM2: T309G, rs2279744 USP7: rs1529916 MUC1: variable number tandem repeat (VNTR) VEGF: G-1154A, rs1570360 TFF3: rs11701143 PTGS2: G-765C, rs20417 PAI-1: rs1799889 eNOS: Glu298Asp, VNTR F2: G20210A, rs1799963 F5: G1691A, rs6025 F8: V34L	None of the following DNA polymorphisms: TP53: rs1042522 LIF: T1414G, rs929271 MDM4: rs1563828 MDM2: T309G, rs2279744 USP7: rs1529916 MUC1: variable number tandem repeat (VNTR) VEGF: G-1154A, rs1570360 TFF3: rs11701143 PTGS2: G-765C, rs20417 PAI-1: rs1799889 eNOS: Glu298Asp, VNTR F2: G20210A, rs1799963 F5: G1691A, rs6025 F8: V34L	-Derived from small-scale studies -Genetic variant expression and its corresponding phenotypes may be specific to certain ethnic groups	(105)
**D Possible non-invasive mechanism-based markers for morphological characteristics of endometrial glands**.				
mRNA levels	Quantitative measurements: CDH1, FOXA2, beta-Catenin, WNT7A, NF2 in glandular epithelial cells WNT7A in luminal and glandular epithelial cells WNT4,11 in luminal epithelial cells WNT16 in stroma cells adjacent to luminal epithelial cells DKK2 in stromal cells on mesometrial side	High expressions of CDH1, FOXA2, beta-Catenin, NF2 in glandular epithelial cells High expression of WNT7a in luminal and glandular epithelial cells High expressions of WNT4, 11 in luminal epithelial cells High expression of WNT16 in stroma cells adjacent to luminal epithelial cells High expression of DKK2 in stromal cells on mesometrial sid	-Only a few animal studies	(106)
Protein levels	Quantitative measurement of MERLIN	High expressions of MERLIN		(107)

#### Validation of Possible Markers

An essential step of validating the clinical value of markers of the WOI is to correlate the markers with the status of the endometrium and live birth rates and other critical clinical outcomes. Key transcriptomic signatures from endometrial cells identified during WOI should be compared with the ultrasound and histological findings of the endometrium. While ultrasound measurements, such as endometrial thickness, have not been concluded as a predictive marker of the optimal WOI, they can be correlated with potential transcriptomic markers of the WOI and histological interpretations to advance understanding of these highly debated markers. Histological findings, such as supranuclear vacuolation and stroma edema, should theoretically correlate with the transcriptomic signatures of the endometrial cells reflective of a receptive status of the endometrium. Pregnancy and delivery outcomes should be monitored and correlated with markers from the molecular test, ultrasound measurements, and histological investigations. Markers of the WOI should be considered valid when the correlation between the markers, the receptive status of the endometrium on histology and live birth, and other clinical parameters is established.

### Roadmap to Develop Proposed Tests for the Optimal Window of Implantation

**Figure 3:**
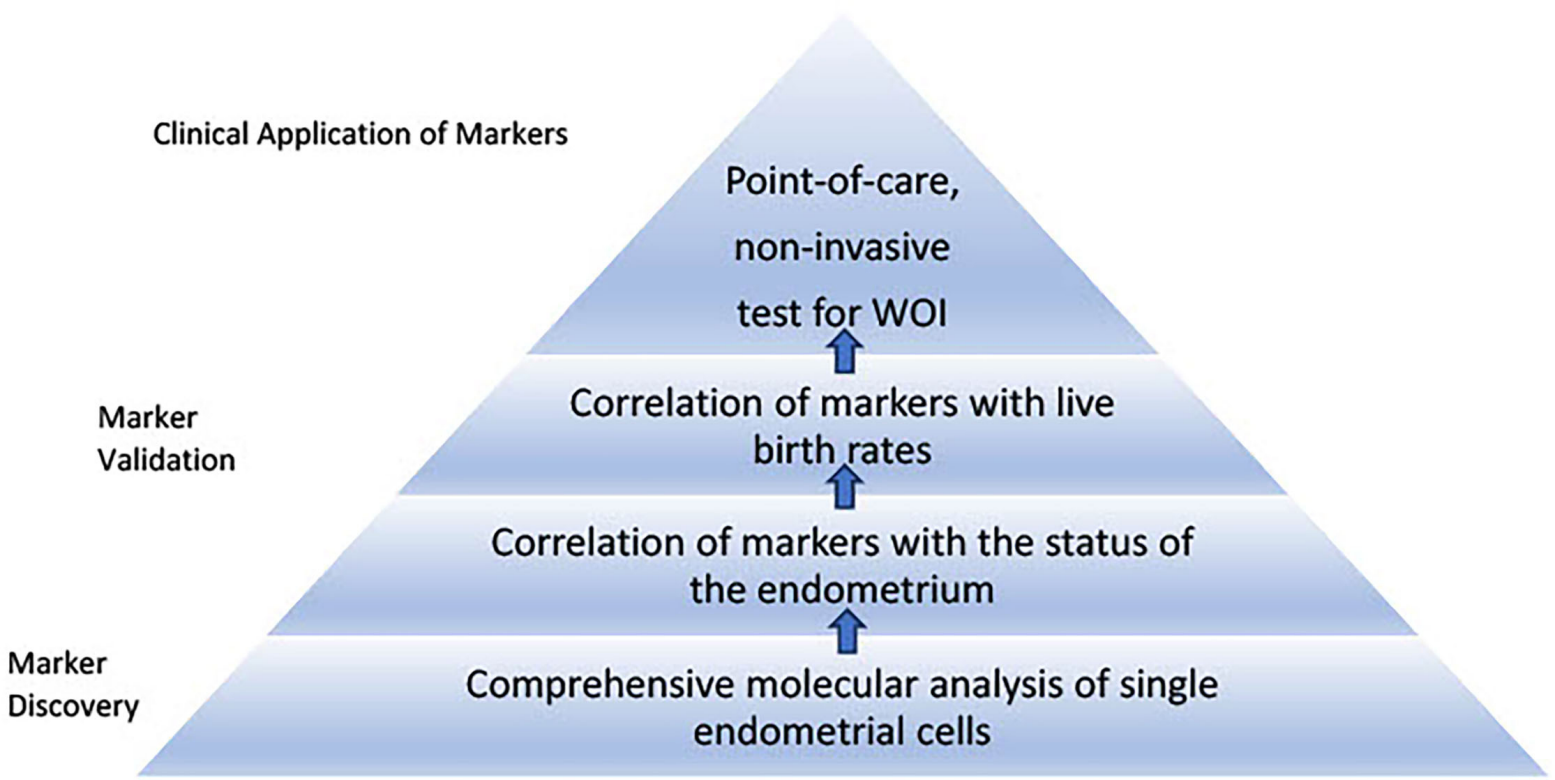
Roadmap to develop proposed tests for the window of implantation. The first stage of the roadmap involves marker discovery through comprehensive single-cell molecular analysis of the endometrial cells. Markers specific to each endometrial cell type associated with histological findings of receptivity and live birth can be validated and thresholds of the markers can be established through large-scale clinical studies. The second stage involves development of point-of-care, non-invasive tests for the window of implantation. This stage requires selection of essential markers from different sources such as blood and urine tests to be integrated into routine clinical use.

Based on the approach of the WOI test development discussed above, a series of stages transitioning from comprehensive single-cell molecular analyses of the endometrium to point-of-care, non-invasive tests using selected validated markers can be imagined (Figure 3). One step in the development of tests for the WOI may involve patients with recurrent implantation failure undergoing ART. A blood test can be drawn at the initial visit to evaluate for the potential genetic variants that impair the endometrial receptivity. An endometrial biopsy could be performed through a mock cycle on the day that an embryo transfer would normally occur. Samples collected from the endometrial biopsy or endometrial fluid would be analyzed through single-cell transcriptomic analysis. Advances in microfluidics offer the technology to develop such tests. For example, an integrated microfluidic chip was developed to capture rare circulating tumor cells from whole blood and obtain single-cell RNA lysates (108). Using the same principles, a point-of-care device can be developed to rapidly enrich, isolate, and identify each endometrial cell as well as to collect target single-cell lysate and conduct single-cell RNA sequencing. The simultaneous single-cell transcriptomics profiling of endometrial cells reveals the molecular dynamics of each cell type and offers an opportunity to uncover suboptimal endometrial receptivity at the molecular level of individual cell populations. For example, thresholds of panels of gene expressions for each type of endometrial cell could be established and assessed during the test. Similarly, thresholds of relative gene expressions among different endometrial cell types and expression levels of essential receptor–ligand pairs can be assessed. If thresholds of marker gene expressions are not met, the test could be repeated the following day. If a trend of markers moving toward the threshold values is recorded, daily tests could be performed until the thresholds of markers are reached. On the other hand, if the thresholds of markers remain unmet, additional investigations, such as metabolomic profiling of the endometrium, may be performed. Daily biopsy of the endometrium is invasive and the procedure itself alters the tissue molecular profile. Therefore, alternative non-invasive methods, such as endometrial fluid or urine sample collection, should be considered. A comprehensive molecular analysis of synchrony among the endometrial cells would stimulate the development of targeted molecular interventions to restore any persistent asynchronous activities. This initial stage of the molecular assessment of the WOI of patients with recurrent implantation failure could advance our understanding of the mechanisms regulating the WOI, lead to the discovery of transcriptomic markers at the single-cell level and establish thresholds of these markers that offer diagnostic value.

**Figure 4:**
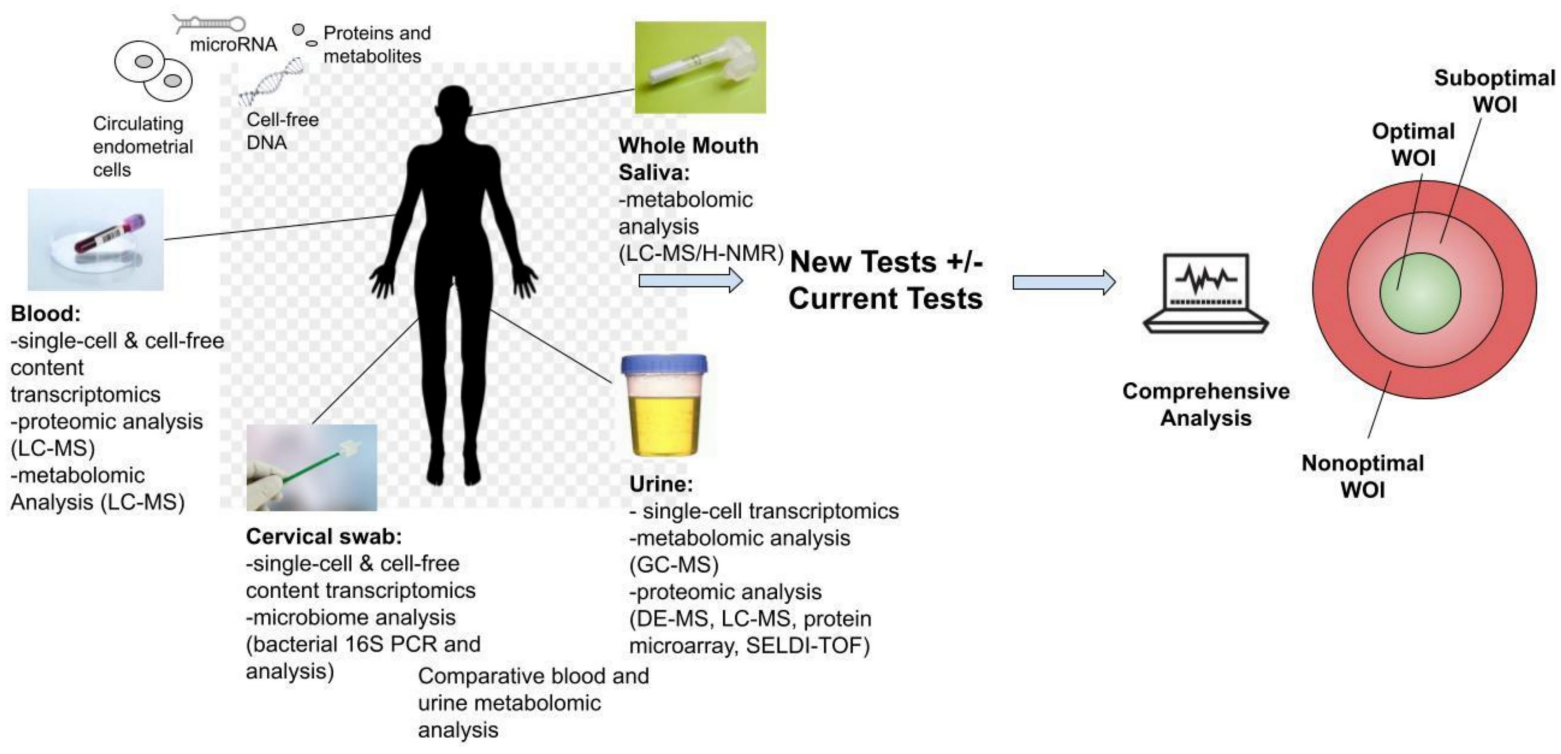
Development of non-invasive tests for the optimal window of implantation (WOI). Multiple analyses could be performed including transcriptomic, metabolomic, proteomic and microbiome profiling through routine saliva, urine, blood or cervical swab tests. The proposed tests above are all currently technically feasible and could be developed into commercial tests. These tests offer the opportunity for advancing the understanding of the biological basis of the endometrial receptivity during the WOI. They also encourage future biomarker discovery, validation and integration of the biomarkers of WOI into daily clinical practice for testing of women of reproductive age to improve pregnancy outcomes.

Another stage of WOI test development is to develop non-invasive, routine tests for all patients seeking ART. While endometrial biopsies offer the most direct route to obtain samples, they are invasive and would not be integrated into routine tests. Non-invasive routes of obtaining samples to perform endometrial receptivity tests, such as blood, urine, saliva, and cervical swab, offer the opportunity for further marker discovery and clinical integration of the test for the optimal WOI (Figure 4). For example, a recent study reported using plasma cell-free RNA signatures obtained from a single blood draw during pregnancy to predict pre-eclampsia and to understand biological processes implicated in the pathophysiology of pre-eclampsia (109). In addition to plasma cell-free RNA profiles, multiple types of biological markers can be analyzed through these non-invasive routes. For example, metabolomic analysis of the saliva and the urine as disease diagnostic biomarkers has been frequently reported (110, 111). A urine metabolomic profiling of the healthy pregnant patients and patients that had a spontaneous abortion revealed significant differences between the two groups. A metabolite panel of indolylacryloylglycine and L-histidine was used in a predictive model for early spontaneous abortion with an AUC of 0.94 (112). Urine metabolic profiles of patients with endometriosis compared to these of health patients showed higher concentrations of N1-methyl-4-pyridone-5-carboxamide, guanidinosuccinate, creatinine, taurine, valine, and 2-hydroxyisovalerate and decreased the concentrations of lysine (113). A recent study of the early mouse embryo development revealed a highly dynamic and interconnected process of metabolic, transcriptional, and epigenetic network remodeling which points to a potentially significant role of metabolic programming in endometrial receptivity (114). The delayed endometrial development and altered progesterone signaling underlying a suboptimal endometrial receptivity may be reflected in the urine metabolome (115). Altered metabolic pathways may be further investigated through differential metabolomic profiles of the urine and the serum (116).

In addition to the metabolomic analysis, single-cell transcriptomic analysis of the endometrial cells can potentially be performed through blood, urine, and cervical swab samples. The challenge of such analysis lies in the detection of endometrial cells in blood, urine, or cervical swab tests. Given the recent progress in high-resolution molecular characterization of endometrial cells and technology for rare cell capture in peripheral blood (9, 117), studies can be performed validating the use of such technology to develop urine, blood, and cervical tests that capture endometrial cells and perform single-cell transcriptomic analysis. Previously, circulating endometrial cells have been isolated in the peripheral blood of women with endometriosis (118–120). The number of these circulating endometrial cells peaked at the mid-secretory phase of the menstrual cycle which corresponds to the decidualization of the uterine lining (121). It is possible that circulating endometrial cells are present in different quantities throughout the menstrual cycle in blood and urine. Intact endometrial cells may be isolated in blood, urine, and cervical swab samples. Adhesion-based microfluidic cell separation systems could potentially capture and isolate these endometrial cell types from the samples (122). And as different biochemical, physical, or electromagnetic properties of these cells are characterized, highly sensitive methods can be developed for their capture and isolation (123). Point-of-care transcriptomic profiling of these captured cells can reveal the molecular asynchrony between the endometrial cells. These distinct transcriptomic patterns present an opportunity for assessment of molecular synchrony between endometrial cells and identification of the opening of the WOI. Furthermore, transcriptomic, proteomic, and metabolomic profiling of the cell-free content as well as microbiome analysis of the cervical samples could be performed as additional sources for potential biomarkers of the WOI. Potential markers from the non-invasive tests should be correlated with the endometrial markers validated in the initial stage.

The value of mechanism-based markers of the optimal WOI extends beyond the diagnostics to the development of interventions to optimize the endometrial receptivity. Many tools, such as optogenetics, now allow clinicians to manipulate the spatiotemporal expression of genes. Takao et al. demonstrated the potential of optogenetic genome editing in fertility medicine using photoactivable Cas9 in living mouse to regulate the mouse embryo implantation (124). Identification and validation of markers and the underlying genetic links to the biological processes implicated in the human implantation process will provide the platform upon which therapeutic interventions using tools, such as optogenetics, can be developed to restore altered WOI.

## Limitations and Challenges of Proposed Pathways for Tests of the WOI

A few limitations and challenges should be addressed for the proposed roadmap of WOI testing. The main limitation during the initial stage of comprehensive molecular analysis of single endometrial cells comes from the quality control process of single-cell RNA sequencing. Current single-cell RNA sequencing technology has a high level of technical noise in the data due to bias of transcript coverage, low capture efficiency, and sequencing coverage (125, 126). Certain transcripts may not be detected using even the most sensitive protocol (127). The capture of low-quality cells, including cells that are dead and mixed with other cells, may lead to misinterpretation of the data (128). Robust quality control methods will enhance the quality of data and lead to more accurate interpretations. In addition, technical variations in the operation of sequencing experiments may lead to that gene expression profile from one batch systematically differing from that in another (129). This batch effect should be corrected in the downstream analysis to avoid masking the underlying biology that regulates the WOI. Another limitation in the development of non-invasive tests of WOI comes from the lack of human studies of WOI-associated markers identified from sources other than an endometrial biopsy. The challenge of identifying the critical markers of WOI through non-invasive routes can only be adequately addressed through a thorough understanding of the underlying biology regulating the WOI. Furthermore, the current cost of single-cell sequencing analysis is 7–15 times the cost of bulk analyses. This high cost presents another barrier for the technology to be integrated into routine clinical use. As the capabilities of the single-cell sequencing methods continue to evolve, the costs will be reduced, making the technology more widely accessible (130).

## Conclusion

Despite the progress made in the understanding of human endometrium physiology and pathophysiology, insufficient progress has been achieved for the integration of clinical tools for prognostic tests and treatments for altered WOI. New strategies in translating our knowledge of the endometrium into clinically useful tests could be used. First of all, the association between the above-mentioned markers and the live birth outcome should be elucidated and supported by molecular mechanisms. In addition, multiple modalities, rather than a single marker, may be used for a comprehensive assessment of the status of the endometrium during the WOI. For instance, endometrial thickness, endometrial receptivity array as well as the proposed blood, urine, saliva, and cervical swab tests mentioned above can be used simultaneously for an evaluation. As sufficient data from test results are obtained and studied with their corresponding live birth outcomes, machine learning methods can be used to help in understanding the critical combination of tests that determine the live birth. And these methods can help to achieve the personalized assessment of the endometrium through prediction of the probably of live birth rate with a given set of inputs of patient information and test results.

Given the detailed molecular characterization of the receptive endometrium and the currently available microfluidic platforms, one can envision performing point-of-care molecular analysis of endometrial cells captured through blood, urine or cervical swab tests and the metabolites captured through urine tests to assess the WOI. In addition, spatial-temporal manipulation of biomarker expressions holds the potential to restore optimal endometrial receptivity during the WOI.

## Author Contributions

All authors listed have made a substantial, direct, and intellectual contribution to the work and approved it for publication.

## Conflict of Interest

The authors declare that the research was conducted in the absence of any commercial or financial relationships that could be construed as a potential conflict of interest.

## Publisher's Note

All claims expressed in this article are solely those of the authors and do not necessarily represent those of their affiliated organizations, or those of the publisher, the editors and the reviewers. Any product that may be evaluated in this article, or claim that may be made by its manufacturer, is not guaranteed or endorsed by the publisher.
